# Instability of CII is needed for efficient switching between lytic and lysogenic development in bacteriophage 186

**DOI:** 10.1093/nar/gkaa1065

**Published:** 2020-11-19

**Authors:** Iain M Murchland, Alexandra Ahlgren-Berg, Julian M J Pietsch, Alejandra Isabel, Ian B Dodd, Keith E Shearwin

**Affiliations:** Department of Molecular and Biomedical Science, University of Adelaide, Adelaide, SA 5005, Australia; Department of Molecular and Biomedical Science, University of Adelaide, Adelaide, SA 5005, Australia; Department of Molecular and Biomedical Science, University of Adelaide, Adelaide, SA 5005, Australia; Department of Molecular and Biomedical Science, University of Adelaide, Adelaide, SA 5005, Australia; Department of Molecular and Biomedical Science, University of Adelaide, Adelaide, SA 5005, Australia; Department of Molecular and Biomedical Science, University of Adelaide, Adelaide, SA 5005, Australia

## Abstract

The CII protein of temperate coliphage 186, like the unrelated CII protein of phage λ, is a transcriptional activator that primes expression of the CI immunity repressor and is critical for efficient establishment of lysogeny. 186-CII is also highly unstable, and we show that *in vivo* degradation is mediated by both FtsH and RseP. We investigated the role of CII instability by constructing a 186 phage encoding a protease resistant CII. The stabilised-CII phage was defective in the lysis-lysogeny decision: choosing lysogeny with close to 100% frequency after infection, and forming prophages that were defective in entering lytic development after UV treatment. While lysogenic CI concentration was unaffected by CII stabilisation, lysogenic transcription and CI expression was elevated after UV. A stochastic model of the 186 network after infection indicated that an unstable CII allowed a rapid increase in CI expression without a large overshoot of the lysogenic level, suggesting that instability enables a decisive commitment to lysogeny with a rapid attainment of sensitivity to prophage induction.

## INTRODUCTION

**Figure 1.**
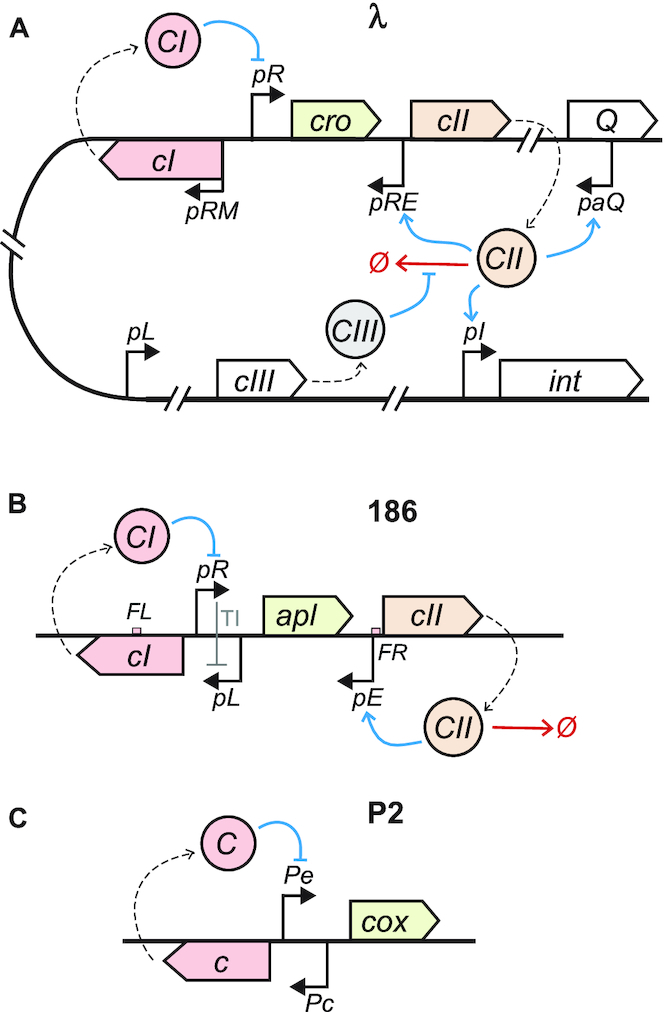
Schematic diagram of the switch regions of the temperate bacteriophage (**A**) λ; (**B**) 186; (**C**) P2. In all cases, rightward transcription represents lytic transcripts, while leftward transcription represents lysogenic transcripts. In (**B**), TI indicates transcriptional interference of *pR* on *pL*, while *FL* and *FR* indicate flanking sites for CI binding.

The genetic and molecular machinery that governs the lytic/lysogenic life cycle decision of temperate phage has proven to be fertile ground for analysing the operation of genetic switches (1,2). The switch region of the λ phage in particular has yielded many insights into the principles and phenomena that underpin the effective function of a bistable switch (1,3–5). The λCII protein is a critical component of the decision-making circuit, being a pro-lysogenic factor necessary for establishing lysogeny after infection. λCII primes production of the lysogenic repressor CI, inhibits expression of late lytic genes and activates expression of the integrase gene (1,6) (Figure 1A). λCII is encoded on the lytic transcript, thus creating a delayed negative feedback on lytic development. λCII is rapidly degraded *in vivo* by the protease FtsH, and is protected from FtsH by the λCIII protein (7–9).

The developmental switch of phage 186 has a very similar topology, encoding a proteolytically degraded, pro-lysogenic factor CII on the lytic transcript (10) (11) (Figure 1B). Yet phage 186 belongs to the P2-related family of bacteriophage, and is evolutionarily distinct from λ, with analogous factors from the two phage typically possessing very little sequence homology (12). The developmental switch from 186-related temperate phage P2 lacks a CII-like factor and the associated delayed negative feedback (13) (Figure 1C), demonstrating that a CII-like factor is not a necessary component of a genetic switch governing two alternative, stable states.

From an evolutionary perspective, we would therefore expect the presence of *cII* in the genetic switch to correlate with additional or improved functionality of the switch in some way. Additionally, the coincidence of proteolytic degradation of both λCII and 186-CII suggests a functional or evolutionary constraint on the half-life of CII. It is tempting to speculate that a rapidly degraded CII may be related to the SOS-inducible phenotype of 186 and λ, which P2 lacks (13–16). Yet the link between genotype and phenotype is not readily apparent, and the overall functionality imparted by CII on this class of genetic switch remains poorly described.

Studies of λ*cII* and λ*cIII* mutants have suggested that proteolysis of λCII affects the relative frequencies with which different developmental fates arise, resulting in changes to the phage frequency of lysogeny (7–9). However the complexity of the phage λ genetic network does not allow this effect to be directly linked to the behaviour of the core developmental switch (*cI*-*pRM-pR-cro-pRE-cII*), since λCII is also active at pro-lysogenic promoters *paQ* and *pI* (17), outside the switch region (Figure 1A). It is therefore unclear whether the loss of λ plaque formation in response to λCII stabilisation (8) is a result of changes in the fate of the switch, or whether the activity of λCII at other promoters disrupts the lytic pathway in other ways. The 186 developmental switch, on the other hand, presents a substantially simpler system for the functional analysis of CII, possessing only a single CII-activated promoter (*pE*), and no CIII-like factor (18).

We have observed a renewed interest in phage biology as a rich source of components for use in synthetic biology, and an associated desire to understand how the properties of individual components affect the emergent properties of small reusable genetic networks. In this work, we seek to characterise the proteolysis of 186-CII and its functional consequences in an effort to further understand the role of CII and its degradation in the 186 switch, and in switches with similar delayed negative feedback topologies.

## MATERIALS AND METHODS

### Strains and constructs

A8295 = W3110 *sfhC zad-220::Tn10*, and A8296, its *ΔftsH3::kan* (19) derivative ( = AR3291 and AR3289 of (20)), were obtained from Amos Oppenheim. KK211 is a *ΔrseP::kan* derivative of AD1811 *Δpro-lac thi ΔrseA::cat* /F’*lacI*^q^*Z*M15 *Y*^+^*pro*^+^ (21), obtained from Y. Akiyama (Kyoto University). KK372 is a *ΔdegS::tet* derivative (21) of CU141 *araD139 Δ(argF-lac)_U169_ rpsL150 relA1 flbB5301 deoC1 ptsF25 rbsR*/F’lacIq Z^+^Y^+^ pro^+^ (22), obtained from Y. Akiyama (Kyoto University). Strains used for reporter assays were derivatives of *Escherichia coli* strain BW25113 *lacIrrnB*_T14_ Δ*lacZ*_W316_*hsdR514* Δ*araBAD*_LD78_ (23). The *cII*^–^ mutation was V36E/Q37S in the helix-turn-helix DNA-binding motif (10). The *apl^–^* mutation was E28R/R29E/R33Q in the helix-turn-helix DNA-binding motif. The *pE^–^* mutation was KS11 (24).

CII expression plasmids pZS45-CII169 (10) and pZS15-CII169 are pSC101 origin plasmids carrying spectinomycin or ampicillin resistance, respectively. Both express CII169 under the control of the wildtype *lac* promoter (with *O3* and *O1*) and the ribosome binding site from pET3a. pZE15-CI.CTD is pZE15-CI.CTDα, a colE1 origin plasmid carrying ampicillin resistance and expressing the 186 CI-CTD fused to the LacZα fragment (25), with promoter and RBS as for pZE15-CII169.

To make the 186 switch region reporter, 186 DNA fragments (*cII*^+^, *cII*^–^ or *cII145*) extending from the left end of the *cI* gene to the start of the *fil* gene (downstream of *cII*) were cloned into pIT3-CL*lacZ*trimfuse (26) such that the first 3 codons of *fil* are fused to the 9th codon of *lacZ(O2^–^)*. The plasmids were integrated into the λ*att* site. The leftward switch reporter was made the same way except the 186 fragments are reversed, extending from the end of the *cII* gene, with codon 9 of *int* (downstream of *cI*) fused to codon 9 of *lacZ(O2^–^)*. The *tum* induction module contains a wildtype 186 *p95.tum* fragment (Genbank: U32222.1 pos. 28984–29538) cloned into pIT3-TO (26) and inserted at the primary 186*att* site (27).

The *pE* reporter was pIT3-CL-*pE.lacZ* (10) integrated into the λ*att* site. The *pR.apl.cII* expression module contained 186 DNA (*apl^+^/apl^–^*, *pE^+^/pE^–^*) from codon 10 of *cI* to the end of *cII*, inserted into pIT3-TH (tetracycline resistant) and integrated into the HK022 *att* site.

### Construction of phage mutants

186.*CmR* was constructed by insertion of a chloramphenicol resistance cassette into the sequence between *cos* and *tW* by recombineering with pKD46 (23). Construction of *cII145*. and 186.*cII^–^* mutants used a two-step recombineering protocol (28). In the first step, the *cII* gene of a wildtype 186 prophage was replaced with a *cII145* or *cII^–^* gene and a selection cassette containing both a chloramphenicol resistance gene and superfolder GFP. In the second step, the selection cassette was deleted from the prophage by recombineering with a single-stranded DNA oligonucleotide. Colonies were screened for a loss of GFP expression. Two independent *cII145* lysogens were obtained and the sequence of the entire switch region from *int* to *fil* confirmed. *cII145* and *cII^–^* phage were made CmR using pSIM6-based recombineering (29) using the same chloramphenicol resistance cassette described above.

### *In vivo* degradation

*In vivo* CII degradation assays were conducted as previously described (10), with pZS45-CII169-containing cells grown with 50 μg/ml spectinomycin and 100 μM IPTG to mid-log phase, and translation inhibited with chloramphenicol (100 μg/ml). When using the pZS15-CII169 plasmid to express CII, cells were grown with 100 μg/ml ampicillin and 100 μM IPTG, and translation was stopped using 200μg/ml spectinomycin.

### Yield of lysogens

Yield of lysogens (the number of lysogens per added phage) was measured according to (30), where addition of 186*cI10* phage kills any non-lysogenic bacteria, but allows lysogens to grow.

### Frequency of lysogeny

Frequency of lysogeny experiments were carried out using phage carrying chloramphenicol resistance as previously described (31), except that media were not supplemented with MgSO_4_ or maltose, and indicator cultures were not concentrated prior to infection. Adsorption was 90–95% efficient. A strain harbouring the pZE15-CI.CTD plasmid was used as an indicator for infectious centre and free phage assays, with growth and plate media supplemented with ampicillin to 100 μg/ml and IPTG to 200 μM.

### UV induction assays

CmR lysogens were cultured overnight at 37°C in M9 minimal media containing 0.5 mM glucose, supplemented with 10 μM Fe(SO_4_)_2_ and 10 μg/ml chloramphenicol. Following overnight growth, cultures were supplemented with 20% glucose to a final glucose concentration of 20mM, and growth at 37°C was continued to log phase. Log phase cultures were diluted 1/1000 into fresh M9 minimal media (20 mM glucose) + 0.1% TWEEN-20, and 5 ml was UV irradiated (0.13 W/m^2^) uncovered in a 90 mm Petri dish for up to 4 min. Prior to and following UV treatment an aliquot was plated on LB agar to assay cell density and survival.

For plaque detection, irradiated cultures were added to BW25113 indicator and 0.7% top agar and immediately plated on TB agar. For lysogen detection, irradiated cultures were added to log phase cultures of the spectinomycin-resistant indicator BW25113 (pIT-SL), and infection was allowed to occur over 10 min. Lysogens were detected by plating on dual-selective LB agar media containing 10μg/mL chloramphenicol and 20 μg/ml spectinomycin.

### CI-CTD induction assays

Cultures of lysogens harbouring the pZE15-CI.CTD and pUHA-1 plasmids were grown overnight at 37°C in LB supplemented with 100 μg/ml ampicillin and 50 μg/ml kanamycin. Overnight cultures were collected by centrifugation and washed twice with an equal volume of fresh media to remove phage particles that may have accumulated due to spontaneous induction. Cultures were diluted to OD_600_ 0.20 in LB + 100 μg/ml ampicillin, 50 μg/ml kanamycin and 200 μM IPTG and cultured at 37°C for 2 h. Phage stocks were prepared from these cultures by centrifugation and treatment with chloroform, and used to infect log-phase cultures of the indicator BW25113 (pIT-SL). Infection was allowed to occur over 10 minutes, before infection mixtures were plated on dual-selective LB agar media containing 10μg/mL chloramphenicol and 20μg/mL spectinomycin to select for lysogens.

### LacZ assays

Kinetic LacZ assays in 96-well microtitre plates were performed as previously described (32).

### UV timecourse

Switch reporter strains were cultured overnight at 37°C in M9 minimal media containing 0.5 mM glucose, supplemented with 10 μM Fe(SO_4_)_2_, 10 μg/ml chloramphenicol and 4 μg/ml tetracycline (for the *cI* transcription reporter, overnight growth was in 1 mM glucose and tetracycline was omitted). Following overnight growth, cultures were supplemented with 20% glucose to a final glucose concentration of 20 mM, diluted to OD_600_ 0.15, and growth at 37°C was continued to early log phase (OD_600_ 0.30–0.40). 5 ml culture + 0.1% TWEEN-20 as a wetting agent was transferred to a 90 mm plastic Petri dish and an aliquot was taken and stored on ice. The culture was UV-irradiated (0.13 W/m^2^) uncovered for 40 s, and returned to a flask for continued incubation with shaking at 37°C. The culture was occasionally diluted during longer incubations to maintain log phase growth. Aliquots were removed from culture at intervals, OD_600_ measured, and stored on ice for LacZ assay. For experiments conducted over 5 h, the experimental culture was diluted to OD_600_ 0.30 and an aliquot was pelleted and stored at −20°C every hour. Cell pellets were subsequently analysed by western blot.

### Mathematical modelling

Simulations were conducted using a modified Gillespie algorithm that maintains well-synchronised discrete and continuous versions of each state variable to enable inter-related deterministic and stochastic calculations to take place (33). The hybrid algorithm numerically integrates deterministic equations between each stochastic event, and updates the cumulative hazard for stochastic events according to a time-dependent hazard function. The differential equations and hazard functions governing our model are described in Supplementary Table S1. Variants of the wildtype 186 genetic circuit were modelled by altering model parameters, as described in Supplementary Table S2.

Transcripts were modelled explicitly, and in the absence of data regarding degradation rates, were assumed to have mean lifetimes similar to the global average for *E. coli* (34). Further details of the mathematical modelling and fitting procedure are included in the Supplementary Data.

## RESULTS

### 186-CII is degraded by FtsH

**Figure 2.**
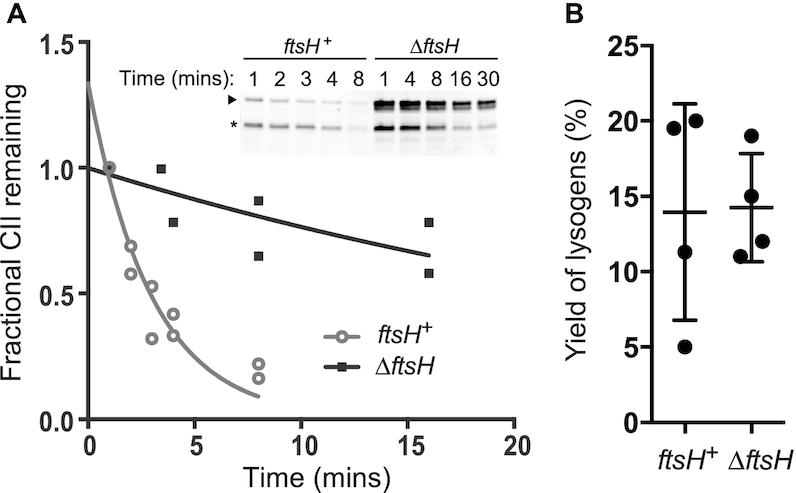
CII is degraded by FtsH. (**A**) *In vivo* degradation assays using A8925 (*ftsH^+^*) (lanes 1–5) or A8926 (*ΔftsH*) (lanes 6–10) expressing CII from pZS45-CII169 with translation inhibited by 100 μg/ml chloramphenicol at time –1 min. Full-length CII is indicated by the arrow; the major degradation product by an asterisk. Volumetric analysis of the full-length CII band shows that the host lacking *ftsH* degrades CII more slowly than the parental strain. Points represent individual measurements, lines show best fit of a one phase exponential decay to a plateau constrained to *y* = 0. Best-fit (95% CI) values for half-lives were 2.1 (1.5–3.1) min for *ftsH^+^* and 25.9 (14.0–180.0) min for *ΔftsH*. (**B**) 186+ was used to measure the yield of lysogens from *ftsH^+^* and *ΔftsH*. Data points represent independent experiments, lines show mean and standard deviation of the ensemble.

We have previously shown that 186-CII is rapidly degraded *in vivo* by an unknown protease (10). λCII is known to be degraded by the *E. coli* protease FtsH/HflB (7), and strains with mutations in *ftsH* yield plaques with increased turbidity relative to parental strains when plated with λ phage (7,8). We therefore hypothesized that FtsH may also be responsible for degradation of 186-CII. To test this hypothesis, we investigated the *in vivo* half-life of CII in the *ΔftsH* strain A8926, and the parental strain A8925 using Western blot analysis following addition of a translation inhibitor. CII degradation results in the formation of a specific, inactive product of proteolysis, corresponding to the first 135 amino acids of the protein (Figure 2A) (10). In the *ΔftsH* strain, this degradation product was still evident, along with an additional degradation product migrating at an apparent molecular weight in between that of full-length CII and the previously characterised degradation product. Nonetheless, quantitative analysis of the full-length CII band shows that degradation is much slower in the *ΔftsH* strain than the control strain (Figure 2A), leading us to conclude that CII is degraded by FtsH.

We further sought to evaluate the effect of stabilisation of CII on the behaviour of the 186 developmental switch. Given the pro-lysogenic function of CII, and the apparent high frequency of lysogeny phenotype of λ in mutants of *ftsH*, we expected that 186 would also exhibit a high yield of lysogens in a *ΔftsH* strain. However, our results show no statistically significant difference between the yield of 186 lysogens in strains A8926 and A8925 (*n* = 4; *P* = 0.93; *t* = 0.075, df = 6; unpaired, two-tailed *t*-test) (Figure 2B). Consistent with this finding, there was also no observable difference in 186 plaque morphology on the two strains.

### 186-CII is degraded by RseP

Since degradation products of CII are still evident despite deletion of *ftsH*, we reasoned that other proteases may also degrade CII. Search approaches exploiting random transposon-based knockouts (35) or using knockouts of non-essential proteases from the Keio collection (36), and screening for changes in CII transcriptional activity or 186 plaque morphology, did not successfully identify additional proteases that degrade CII. A limitation of both approaches is that the role of proteases essential for host function cannot be examined. This explains why FtsH was not identified as a CII-degrading enzyme by these experiments, since *ftsH* deletion alone is lethal to the host; the *ΔftsH* strain A8926 we used carries the additional mutation *sfhC21*, which suppresses lethality of *ΔftsH*.

**Figure 3.**
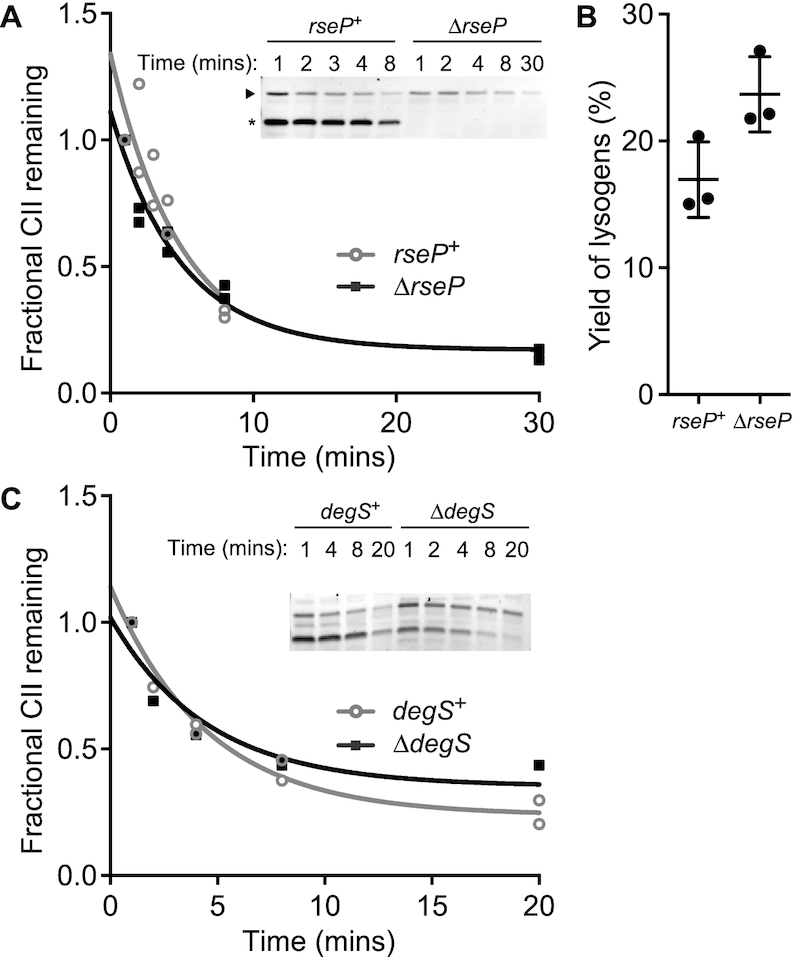
CII is degraded by RseP. (**A**) *In vivo* degradation assays using AD1811 (lanes 1–5) or KK211 (lanes 6–10) expressing CII from pZS15-CII169 with translation inhibited by 200 μg/ml spectinomycin at time –1 min. Volumetric analysis of the full-length CII band from *in vivo* degradation assays shows that degradation of full-length CII is not significantly slower in hosts lacking *rseP*. (**B**) 186+ was used to measure the yield of lysogens from AD1811 and K211. Data points represent independent experiments, lines show mean and standard deviation of the ensemble. Probability of no difference between means = 0.0505, unpaired *t*-test, *n* = 3. (**C**) Translation stop *in vivo* degradation assays using CU141 (lanes 1–5) or KK372 (lanes 6–10) expressing CII from pZS15-CII169.

We therefore sought to determine whether DegS or RseP (formerly YaeL) degrade CII, since single-gene deletions of these proteases are also lethal to the host (21). Again taking advantage of additional mutations that suppress lethality of protease deletions, we investigated the *in vivo* degradation of CII in the *ΔrsePΔrseA* strain KK211 and the parental *ΔrseA* strain AD1811. Using Western blot analysis after addition of a translation inhibitor, we found that deletion of *rseP* resulted in the loss of the CII degradation product (Figure 3A). Thus, we conclude that RseP degrades CII, in addition to FtsH. Quantitative analysis of the full-length CII band does not show evidence that degradation of CII is slowed by genetic deletion of *rseP* (Figure 3A), presumably due to redundancy in degradation of CII via the FtsH pathway. Direct experimentation to confirm this hypothesis was not possible, due to the fact that a dual *ΔrseP/ΔftsH* knockout is not viable (37).

Yield of lysogen experiments suggest that deletion of *rseP* results in a slight (40%) increase in 186 lysogenisation (Figure 3B). Such a result is unexpected, given that we see no detectable stabilisation of CII in the Δ*rseP* strain, yet stabilisation of CII in the *ΔftsH* strain is not associated with a change in the yield of lysogens. Translation-stop *in vivo* degradation experiments using strain CU141 and its *ΔdegS* derivative KK372 show no increase in CII half-life, nor loss of the CII degradation product (Figure 3C). Thus, we conclude that CII is not degraded by DegS.

Our conclusion from this set of results is that the use of protease deletion strains is an unreliable method of probing the effects of stabilising CII on the behaviour of the 186 switch. Both FtsH and RseP are involved in regulating stress response pathways to which 186 may have evolved to respond. We and others (20,37) have observed that the deletion strains of the essential proteases *ftsH* and *rseP* exhibit substantially different growth rates and temperature sensitivity to their parental strains. Furthermore, both FtsH and RseP are responsible for processing sigma factors (21,38), making us sceptical that the intracellular environment—particularly the transcriptional machinery that is central to the switch's function—is comparable between the protease deletion strains and their respective parental controls.

### Poor prophage induction of a 186 with a stabilised CII

Therefore, to better understand how reducing the rate of degradation of CII changes the behaviour of the 186 developmental switch, we stabilised CII by alteration of the protein, rather than by alteration of the host. We have previously shown a C-terminal truncation of CII that retains the first 145 residues, termed CII145, to be stabilised yet retain the same specific activity as the full-length CII protein (10). Thus we created a mutant of the 186 phage, *cII145*, encoding the stabilised CII145 truncation of CII, using recombineering methods to alter a 186 prophage. Two independent *cII145* phage were created and characterised. We also created the chloramphenicol resistant phage variants 186^+^.CmR, *cII^–^*.CmR and *cII145*.CmR, which carry a chloramphenicol acetyltransferase expression cassette in the untranscribed *cos* region of the 186 genome (position 126).

**Figure 4.**
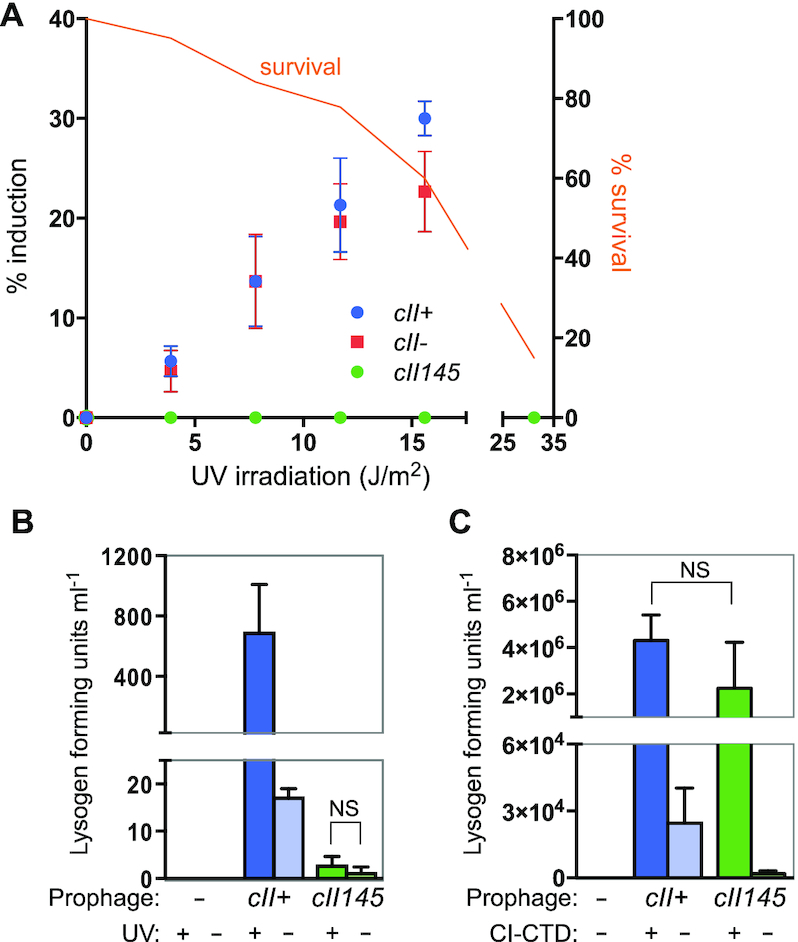
Induction of 186 prophage. Error bars are standard deviations. (**A**) Induction of 186^+^.CmR, *cII^–^*.CmR and *cII145*.CmR prophages by UV irradiation and detection of plaque-forming units. % induction (left axis) is defined as number of plaques induced/number of input cells. Cell survival (right axis) is defined as number of colony-forming units following irradiation/number of input cells. (**B**) Induction of 186^+^.CmR and *cII145*.CmR lysogens by UV irradiation (15.6 J/m^2^) and detection of lysogen-forming units. * signifies *P* < 0.05; ** *P* < 0.01; *** *P* < 0.001 using an unpaired, two-tailed *t*-test (*n* = 3 for *cII^+^* and 6 for *cII145*). (**C**) Induction of 186^+^.CmR and *cII145*.CmR lysogens by expression of CI-CTD (vs empty plasmid controls) and detection of lysogen-forming units. Note that a more dense culture was used for induction compared to (**B**). Statistics as in (B), except *n* = 4.

In order to characterise the phenotype of the *cII145* phage, we first compared induction of *cII145*.CmR, 186^+^.CmR and *cII^–^*.CmR lysogens by UV irradiation. Assaying for induction by the formation of plaques on a lawn of indicator after UV irradiation of lysogens, *cII145*.CmR did not show any sign of induction, while no consistent difference was observed between induction of 186^+^.CmR and *cII^–^*.CmR (Figure 4A). There are two possible explanations for this result. The first is that *cII145*.CmR simply cannot be induced to form viable daughter phage, while the second is that *cII145*.CmR induces, forms viable daughter phage, but cannot form plaques. Investigating the latter possibility, we again attempted to induce the phage (or a non-lysogen control) by UV irradiation, this time using a spectinomycin-resistant indicator strain, and selecting for lysogens that are the product of these infections by dual-resistance to chloramphenicol and spectinomycin. Figure 4B shows that both spontaneous and UV-stimulated induction of 186^+^.CmR is detectable by this method. The data also reveal that *cII145*.CmR induces spontaneously at a low level, and that this is not significantly increased by UV irradiation. However, the number of lysogen forming units (lfu) detected after irradiation of *cII145*.CmR is ∼200-fold lower than that of 186^+^.CmR, suggesting that induction is compromised in *cII145*.CmR. It is important to note that due to the design of this assay, we cannot be confident of the magnitude of this defect from this data alone, given that the number of lysogen forming units is affected by other characteristics of the phage, such as its frequency of lysogeny.

Having established that *cII145*.CmR can be induced and produces infective virions, but with low efficiency, we used an artificial, more efficient method of induction which avoids the use of potentially mutagenic UV irradiation to create phage stocks of *cII145*.CmR for further experimentation. The method uses IPTG-induced over-expression of the dominant-negative C-terminal domain of the 186-CI lysogeny maintenance repressor (CI-CTD) to sequester wildtype CI in the lysogen, and de-repress the lytic promoters. Analysing the efficiency of this form of induction reveals no significant deficit in the number of lysogen forming units detected after induction of the *cII145*.CmR prophage compared to *cII^+^* (Figure 4C). Again care must be taken in the interpretation of these results due to the likelihood of differences in the frequencies of lysogeny of the two phages.

### cII145 frequency of lysogeny

Using the *cII145*.CmR and 186^+^.CmR phage, we investigated how stabilisation of CII affects the frequency of lysogenisation after phage 186 infection. Frequencies of lysogeny were calculated by assaying an infection for the formation of lysogens by chloramphenicol resistance, for plaque formation, and for efficiency of infection. Consistent with the results of our induction experiments, we observed lysogens but no plaques due to infection with *cII145*.CmR, and thus derive a frequency of lysogeny of 100% for *cII145*.CmR, compared to ca. 10% for 186^+^.CmR (Table 1).

**Table 1.** Frequencies of lysogeny of 186 variants

	Host	
Phage	BW25113	BW25113 pZE15-CICTD
186^+^.CmR	9.64 ± 2.13%	0.03 ± 0.07%
*cII145*.CmR	100 ± 0%	58.3 ± 3.13%

Data show mean ± standard deviation of *n* ≥ 4 experiments.

Without proof that *cII145*.CmR is competent to produce a plaque, however, this result has multiple interpretations. It is possible that stabilisation of CII inhibits lytic development itself (for instance by transcriptional interference between *pR* and *pE*), rather than solely influencing the fate of the developmental switch. In this scenario, the true rate at which the switch resolves into the lysogenic state may be substantially lower than that revealed by experiment, but would be masked by ‘abortive lytic’ development, in which infections proceeding down a lytic path do not successfully produce a burst of daughter phage, thus yielding neither plaque nor lysogen.

Addressing this possibility, we repeated our frequency of lysogeny determination, but using a strain expressing CI-CTD as the indicator. Infections of this host should be directed more often to a lytic fate due to the dominant-negative action of CI-CTD (25) against the lysogeny maintenance repressor. Our results match this prediction exactly (Table 1). More importantly, using this strain we observed plaques of *cII145*.CmR, showing that the activity of CII145 itself does not prevent plaque formation, and suggesting that *cII145*.CmR does not undergo abortive lytic development (Table 1).

### Understanding the prophage induction impairment of cII145

**Figure 5.**
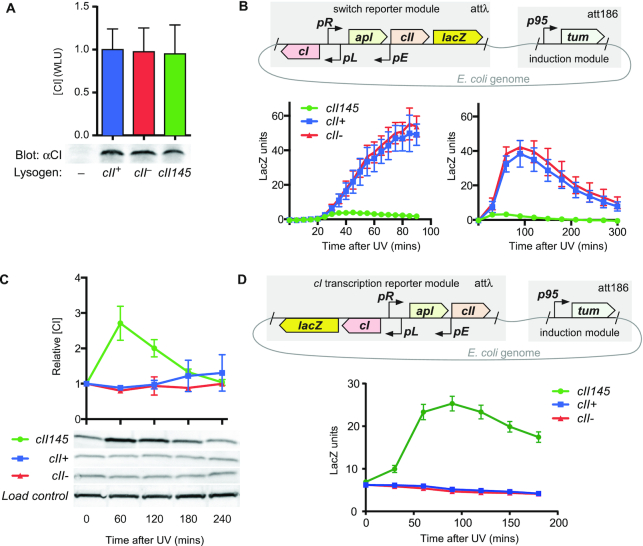
Regulatory effects of CII stabilization. (**A**) Lysogenic CI concentrations of 186 variants. Relative levels of CI present in non-lysogens and lysogens of 186^+^.CmR, *cII^–^*.CmR and *cII145*.CmR as detected by Western blot. Bars show mean and standard deviation (*n* = 4) of quantitation of the CI band (expressed relative to 186^+^.CmR) for experimental repeats. There was no statistically significant difference between CI levels in lysogens of 186^+^.CmR and *cII^–^*.CmR (*P* = 0.90; unpaired, two-tailed *t*-test), nor 186^+^.CmR and *cII145*.CmR (*P* = 0.82; unpaired, two-tailed *t*-test). (**B**) Timecourses of *pR* activity after UV induction. The schematic shows the switch reporter and the 186 *tum* induction module. The *lacZ* gene was translationally fused to the 186 *fil* gene. After UV irradiation (5.2 J/m^2^) of strains, *pR* activity was measured by β-galactosidase assay every 5 or 30 min, for 90 or 300 min, respectively. (**C**) Expression of CI was measured by Western blot in the same irradiated cultures used for β-galactosidase assay in (B). Analysis of CI Western blots shows a significant elevation of CI in the *cII145* variant following UV induction, but not in the *cII^+^* or *cII^–^* variants. Plots in (**B**) and (**C**) show mean and standard deviation (*n* = 3). (**D**) As in (B), except the *lacZ* gene was a translational fusion to the 186 *int* gene in order to measure leftward transcription. Errors are 95% confidence limits (*n* = 6–8).

Having demonstrated that stabilisation of 186-CII results in an impairment of induction and an elevated frequency of lysogeny, we sought to explain the mechanism(s) by which these changes occur. In particular, impaired induction has multiple possible explanations. The simplest is that low, or ‘leaky’ levels of transcription from *pR* in the lysogenic state allows accumulation of CII145, in contrast to a wildtype prophage in which CII would be cleared by proteolysis. A prediction of this model is that on average, lysogens of *cII145*.CmR would have higher lysogenic concentrations of CI due to *pE* activity (Figure 1). Examining this using quantitative western blot analysis, we find that there is no detectable change in relative lysogenic concentrations of CI in lysogens of *cII145*.CmR nor *cII-*.CmR relative to 186^+^.CmR (Figure 5A).

### Non-equilibrium effects of 186-CII stabilisation

Since the equilibrium lysogenic concentration of CI is unable to explain the impairment of *cII145*.CmR prophage induction we investigated the non-equilibrium effects of 186-CII stabilisation through the construction and characterisation of a minimal synthetic switch and *lacZ* reporter, integrated into the *E. coli* genome (Figure 5B). Induction of 186 occurs via expression of the anti-CI factor Tum (15,16), due to SOS mediated de-repression of the LexA-regulated *p95* promoter. Thus integration of a *p95-tum* expression module alongside a switch construct allows investigation of the UV-induction process through reporter assays.

Following exposure to UV, expression of the switch reporter (Figure 5B) was assayed over the ensuing 5 h. For the wildtype construct, we expect that UV irradiation will induce expression of the antirepressor Tum, and a consequent de-repression of *pR* through Tum inhibition of CI. Our results show that de-repression of *pR* in response to UV irradiation is also detectable in the *cII*145 variant of the construct, but is of much lower magnitude than in the *cII*^+^ or the *cII*^–^ variants (Figure 5B). Repression of *pR* is re-established once the SOS signal decays and *tum* becomes again repressed by LexA. This re-establishment of *pR* repression is faster in the *cII*145 variant.

We expected that the behaviour of the switch reporter in the *cII145* variant is due to high expression of CI caused by high levels of CII-stimulated *pE* activity. Accordingly, observing the evolution of CI levels over time by Western blot, we found strongly elevated CI in the *cII145* construct following induction (Figure 5C). As a further test, we placed the *lacZ* gene at the left end of the switch construct to measure transcription of *cI* from *pL* and *pE* (Figure 5D). This showed a large pulse of *cI* transcription in the *cII145* variant, while the activity of the stable LacZ protein fell slowly in the *cII*^+^ or and *cII*^–^ variants. These results indicate that CII stabilisation reduces de-repression of *pR* after UV induction due to over-expression of CI to levels that exceed the sequestration capacity of Tum. Interestingly, no more than minor differences after induction are seen between the *cII*^+^ and *cII*^–^ variants (Figure 5B–D), showing that in the presence of normal CII degradation the effects of a single copy of the wildtype *cII* gene are effectively nullified.

**Figure 6.**
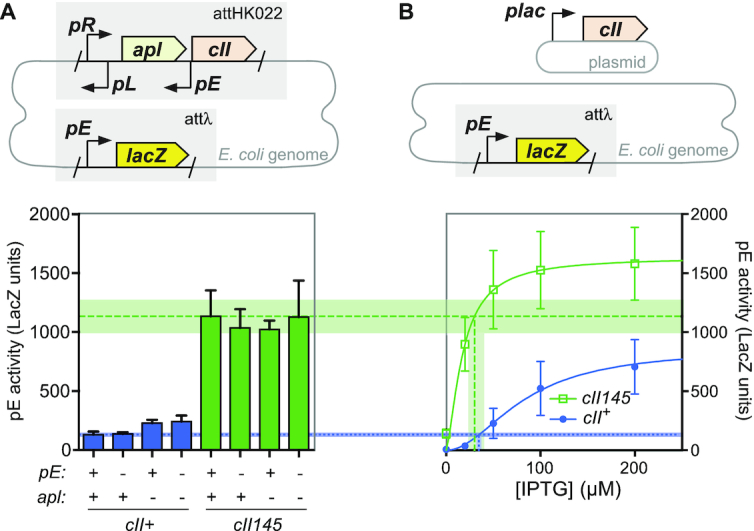
Steady-state CII activity. (**A**) The activity of CII expressed from the *pR* promoter in the absence of CI (upper module) was assayed by a *pE.lacZ* reporter *in trans* (lower module) by β-galactosidase assay at steady state. Bars show mean and standard deviation (*n* ≥ 11). (**B**) The activity of CII expressed with IPTG induction from pZS45-CII169 or pZS45-CII145, assayed by the same reporter, as reported in (10). The plot shows the mean, standard deviation (*n* ≥ 6) and Hill fits.

An alternative, but not mutually exclusive mechanism for reduced de-repression of *pR* is transcriptional interference (32,39). Because *pR* and *pE* are convergent promoters, it is possible that high levels of *pE* activity will reduce transcriptional activity from *pR*. Investigating this hypothesis, we used an integrated switch reporter construct lacking CI, and thus constitutively expressing CII, and reporting CII levels by way of a *pE*-*lacZ* reporter *in trans* (Figure 6A). In this way, reduced *pR* activity can be detected by lower expression of the *lacZ* reporter, while the effect of *pE* activity on *pR* can be determined by mutation of the *pE* sequence in the switch construct. A confounding factor is the presence of the *apl* gene, encoding a weak repressor of *pR*, on the *pR* transcript. To address this, mutants of *apl* that abrogate DNA binding were also included in this experimental design. For a construct encoding wildtype, unstable CII, abrogation of *pE* activity in the switch module has no detectable effect on reporter activity, while as expected, abrogation of Apl activity increases reporter activity (Figure 6A). Using the *cII145* variants, abrogation of neither *pE* nor Apl activity cause a statistically significant variation in reporter activity, demonstrating that even high levels of *pE* activity do not alter *pR* activity via transcriptional interference. We also compared the reporter activities from this experiment with standard curves derived by IPTG-inducible expression of CII or CII145 from a plasmid (Figure 6B) (10). This comparison allows us to calculate a level of IPTG induction (CII expression) that would give rise to the equivalent reporter activity as observed using the switch fragments. This analysis (Figure 6) shows that the IPTG induction levels for CII and CII145 overlap, showing that the activity of *pR* is the same in the *cII*^+^ and *cII145* cases, adding further support to our conclusion that transcriptional interference of *pE* on *pR* is not significant.

### A mathematical model of the 186 switch

Our characterisation of *cII145* shows that stabilising CII both increases the frequency of lysogeny, and prevents induction of the prophage. A theoretical description of the decision-making gene network is a useful complement to these empirical observations to help better illuminate the functional consequences of CII stabilisation. In particular, it allows us to vary the parameters of any regulatory element precisely, without the constraint of what is biologically possible or evolutionarily accessible. Such precision also allows us to incorporate pairs of changes that compensate for each other exactly, in order to preserve key characteristics of the network, while altering parameters of interest. In addition, it gives us ready, reliable and fine-scaled access to information about the dynamics of the network, especially over the short period after infection, which are technically difficult to access experimentally.

Most of the key, individual regulatory elements in the 186 switch region have previously been characterised quantitatively using empirical methods, or parameters describing their behaviour are available from previously published theoretical models. This motivated the construction of a hybrid stochastic-deterministic model of the gene regulatory network depicted in Figure 1B (equations are listed in Supplementary Table S1). The only known phenomena which were not captured in our model of the network were repression of *pE* by CI binding at its flanking *FR* site (11) (Figure 1B), and transcriptional interference. In the case of CI binding at *FR*, a satisfactory quantitative description is not available for implementation. Transcriptional interference between *pR* and *pL* was not modelled explicitly, but a simplified treatment of its effects are inherent in the parameters used to describe CI regulation of *pL* (Supplementary Figure S1). Short timescale events, and events driven by large numbers of molecules (protein–DNA binding equilibria, protein production and degradation) were modelled deterministically, while more sparse events (promoter firing, degradation of transcripts) were simulated stochastically as inhomogeneous Poisson processes according to a hybrid Gillespie algorithm (33).

**Figure 7.**
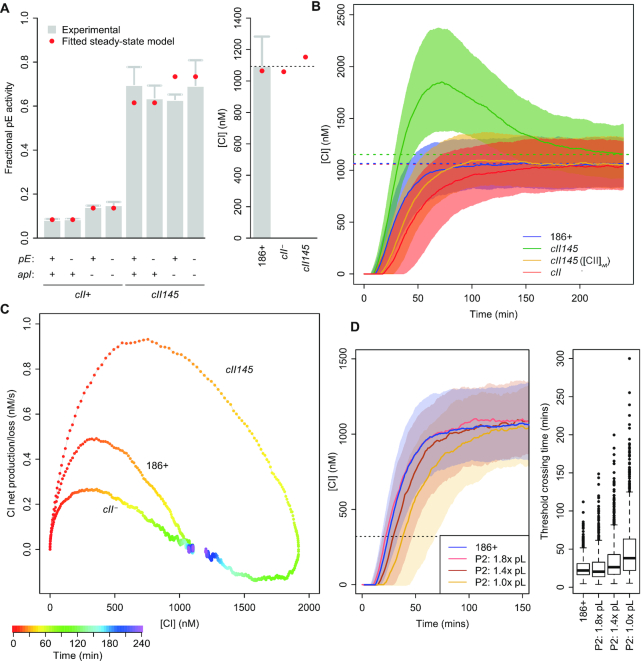
Mathematical modelling of the 186 genetic switch. (**A**) Translation initiation rate parameters of the model were optimised to fit (red circles) the measured *pE* activity of switch variants (left panel) and lysogenic CI concentrations (right panel). Experimental data is the same as that in Figures 6A and 5A; a fractional *pE* activity of 1 corresponds to 1635 LacZ units. (**B**) Plot of the evolution of median CI concentration over time in variants of the 186 genetic switch (*n* = 3000 simulations per curve; shaded area is the interquartile range). Rapidly rising CI concentrations in the stable *cII145* variant explain the high frequency of lysogeny phenotype. Slow increases in and equilibration of CI concentrations in other *cII145* derivatives relative to *cII^+^* suggest a low frequency phenotype for these variants. Dotted lines indicate final lysogenic levels of CI. (**C**) Plot of CI concentration vs net CI production (*n* = 3000 simulations per curve). High maximal CI production in *cII145* provide an explanation for the impaired induction phenotype of this variant. (**D**) *cII^–^* gene network variants can achieve a similar evolution of CI concentrations to that of the wild type 186 network, in the case where *pL* activity is stronger and CI repression of *pL* is enhanced. Each curve shows median CI concentration from *n* = 3000 simulations. The box plots on the right of panel D show the distribution of times at which [CI] = 320nM was reached in the simulations. This [CI] threshold represents 100× the EC_50_ for CI repression of *pR*.

Translation initiation rates have not previously been determined, so the measured lysogenic concentration of CI (40), the relative lysogenic concentrations of CI in variant lysogens (Figure 5), and the *pE-lacZ* reporter construct data presented in Figure 6B were used as constraints to fit these parameters (Figure 7A). Parameter optimisation was made tractable by exploiting the fact that the experimental measurements were all steady-state population averages. After verifying that over a wide parameter range the average behaviour of the stochastic model at steady state closely matched that of an equivalent deterministic model, we could fit the translation initiation rates using steady-state solutions of the deterministic model.

It should be noted that our theoretical model is not expected to exhibit clear bistability - that is, simulated infections based on the model do not yield observable, stable, distinct, ‘lytic’ and ‘lysogenic’ states. Rather, all simulations eventually produce long-run average CI concentrations corresponding to the lysogenic state, but the time taken to resolve into such a state varies between sister infection simulations. Recent experimental work (41,42) suggests that the fate of the switch is not set in a single event, but is a consequence of a cascade of interrelated events. Under this paradigm, only lysogeny need be a genuinely stable state; the lytic cascade ends not in a stable state, but with the destruction of the host cell and release of daughter phage. Thus, the behaviour of our model can be seen as consistent with the absence of later lytic functions or any representation of phage replication in our model.

The mathematical model allowed us to explore the consequences of a wide range of alternative parameters relating to CII function. In addition to the *cII145* (stabilised CII) and *cII*^–^ variants examined in experiments, we are able to simulate networks in which CII is stabilised, but its activity is compromised in different ways, such as weaker binding to *pE* or lower maximal activity of *pE*. In this way, we can interrogate more directly the effects of CII half-life by constructing networks in which the equilibrium activity of *pE* is unaltered from that due to wildtype 186, but the half-life of CII is extended.

### Modelling is consistent with the behaviour of CII145

We used the evolution of CI concentration over time, averaged across a population of simulated infections, as a proxy for the population's progress toward the lysogenic state to investigate the effects of CII stabilisation (Figure 7B). Consistent with expectations and our experimental results, *cII145* exhibited elevated CI concentrations following infection, before eventually equilibrating to a lysogenic CI concentration close to that of the wildtype network. A stabilised *cII* variant with a compensating reduction in production rate (to give steady-state CII levels equal to wildtype) did not give the overshoot in CI levels but gave a slower accumulation of CI than *cII^+^* (Figure 7B), which we predict would correspond with a reduced frequency of lysogeny, as it does for the *cII*^–^ variant.

Another way to view the function of CII in the network is as an additional layer of negative autoregulation of CI production (Figure 1B). CI represses its own production via its action at the *pL* promoter. CI however, also represses CII production (via *pR*), which in turn causes CI production (via *pE*). Therefore, we used the model to simulate infections and examine the relationship between globally averaged CI concentrations and the net rate of CI production/loss for the network as a whole. Analysing the results this way (Figure 7C) reiterates the findings described above that CII145 takes longer to equilibrate and overshoots the lysogenic concentration of CI. Additionally, however, this analysis highlights the maximal rate of net production of CI. The significance of the maximal rate of net CI production is that in order to reduce CI concentrations to near-zero in the process of induction, the phage must produce the CI inhibitor Tum at or above this rate (or in the case of phage λ, induction must accelerate CI degradation by at least this amount). Failure to do so will result in a net accumulation of active CI and result in the phage being pushed back into the lysogenic state.

Figure 7C shows that the maximal CI production rate is >2-fold higher for *cII145* relative to wild type 186. This explains our experimental finding that UV induction is severely impaired, suggesting that only a small fraction of lysogens produce Tum at a sufficient rate to induce the phage after UV irradiation. In contrast, high rates of expression of CI-CTD from a high-copy number plasmid exceed even the *cII145* maximal CI production rate, yielding similar levels of induction to wild type 186.

### Modelling networks without CII

Given our finding that a rapidly degraded CII helps the switch to rapidly reach yet not massively exceed the lysogenic concentration of CI, we sought to investigate whether these properties can be achieved without a CII-like factor, in a network resembling that of P2 (Figure 1). That is, if we assume that faster equilibration to lysogenic CI concentrations is an evolutionary advantage for an inducible phage, can we expect to find inducible P2-like phages that lack a *cII* gene, but preserve the characteristics of *cII*-containing networks that we have identified here?

Without CII present, the *pL* promoter would need to have a higher rate of maximal activity in order to produce CI at the requisite rate. However, simply increasing *pL* activity would also raises the equilibrium concentration of CI. Regulation of *pL* (like the analogous *pRM* promoter in phage λ) has a characteristic biphasic response to CI concentrations, with an activation phase at low [CI] and repression at higher [CI] (40,43). Thus, increasing the strength of *pL* but increasing its repression by CI (through the EC_50_) provides a mechanism for raising *pL* activity without a concomitant rise in equilibrium CI concentration. We adjusted these parameters to give the best fit to the wild type 186 CI concentration curve, finding that, for a *pL* strength of 1.8x, adjustments to the EC_50_ can indeed produce a close match with the average trajectory of the wild type 186 curve (Figure 7D). Thus, we conclude that if faster equilibration of CI concentrations is the principal function of *cII*, then it is at least theoretically plausible for phage to evolve switch networks that lack a *cII*-like factor to achieve the same ends. However, we noticed that the rate of CI accumulation early in infection in the wildtype network was subject to less variation compared to the P2-like networks, shown by the time taken to reach 320 nM CI in different simulation runs (Figure 7D).

## DISCUSSION

We have shown that CII is degraded by at least two essential proteases in *E. coli*, suggesting that maintenance of CII degradation is a strong evolutionary constraint on 186. Our data also show that the 186 yield of lysogens is remarkably resilient to deletions of either one of these proteases. As we have noted above, the nature of *ftsH* and *rseP* protease deletions and their phenotypes lead us to treat these data with some caution. Yet if any inference is to be drawn from these findings, it is that 186 does not use changes in protease abundance, localisation, or activity in the host to sense intracellular conditions and adjust the respective probabilities of the lytic or lysogenic fates, as is suggested to be the case for phage λ (1,44–46).

Nonetheless, stabilisation of CII through deletion of the protease-recruiting C-terminal region yields a clearly demonstrable increase in frequency of lysogeny, and a resistance to induction through over-production of CI following the induction stimulus. Though we have reported a 100% frequency of lysogeny, the true frequency may be slightly lower than 100%. This is because visible plaques will cease to form at a true frequency of lysogeny below 100%, since at high frequencies of lysogeny, insufficient quantities of daughter phage will be produced through lytic development to continue the expansion of a plaque to a visible size.

A tightly specified mathematical model of the 186 switch genetic circuit matched experimental results from this and other studies closely. Our mathematical model can also explain both the increased frequency of lysogeny and impaired induction phenotypes of *cII145*. Simulations of 186 variants suggest that varying only the activity of CII results in a trade-off between quickly rising CI concentrations that enable an early decision upon infection, and fast establishment of a low but stable lysogenic CI concentration that facilitates induction. Fast degradation of a highly active CII reduces the severity of this trade-off, enabling fast-rising and fast-equilibrating CI concentration trajectories. Both of these properties are likely to be crucial in a competitive ecosystem with multiple phages. Fast lytic development is an obvious competitive advantage, but requires a commensurately rapid developmental decision.

Maximal CI production rates from wild type 186 and *cII145* trajectories satisfactorily explain the induction impairment of *cII145*, but falsely predict that 186.*cII^–^* will induce more readily than 186*.cII*+. This could be explained by suppression of CII activity by SOS-induced host factors during induction, in an analogous manner to that observed in phage λ (47), however such a phenomenon has not been tested in 186. One possibility is that inhibition of *pE* by CI binding at *FR* (11) reduces CI production during prophage induction due to CI activity that escapes inactivation by Tum. The λ phenomenon is saturable; over-expression of λCII restores its function during induction (47), which is directly analogous to our data showing CII145 activity following induction.

The results of infection simulations suggest that a key function of a highly active, rapidly degraded CII is to rapidly equilibrate CI concentrations in a lysogen. This has the advantage of ensuring that the lysogen is established and ready for induction as soon after infection as possible. One would expect this to be a more important property for SOS-inducible phages than non-inducible phages, which rely passively on spontaneous induction and so need not prefer an induction-ready state. An alternative mechanism to achieve rapid equilibration of CI concentrations is for CI itself to be rapidly degraded. Others however, have noted that a short CI half-life is likely to be detrimental to the lysogeny-maintenance function of CI, since a short-lived CI is more sensitive to stochastic noise in CI transcription (48).

Modelling of *cII^–^* variants also suggests that rapid equilibration of CI concentrations could also be achieved by increasing *pL* activity, while increasing CI negative autoregulation through *pL* repression. While this is a simple adjustment to make in a theoretical model, the biological accessibility of this parameter space may well be more limited. CI repression of *pL* and *pR* is the product of a high-order multimer binding to five distinct operators (25,49) and increased CI binding to *pL* is likely to also affect *pL* activation and *pR* repression. Thus, the addition of a delayed negative feedback such as the *cII-pE* system to the switch network may represent a more evolutionarily accessible solution. The use of an additional CII-like factor in the network has other advantages. The opportunity to respond differently during infection and induction is one such example, as described above in the case of SOS-induced inactivation of λCII at low concentrations. Our analysis suggests also that the CII-containing network showed reduced variability in the rate of CI accumulation, though whether this would be an advantage for the phage is not clear.

From a synthetic biology circuit design perspective, the use of a CII-like delayed negative feedback similarly presents a simple mechanism to create additional layers of pseudo-autoregulation, without needing to engineer complex promoters. Where the purpose of a CII-like delayed negative feedback is as a form of autoregulation, a short CII half-life is an essential element of this design. With rapid, continuous degradation of CII, the cellular concentration of CII most accurately encodes the immediate past activity of *pR*, and so is a reasonable approximation of current *pR* activity. In contrast, a stable CII results in cellular concentrations of CII that encode *pR* activity averaged over a long window of the past. If the aim is to regulate CI production with respect to its current concentration, then a stable CII builds into the genetic network an unwanted memory of past CI concentrations and *pR* activities.

## Supplementary Material

gkaa1065_Supplemental_File

## Notes

Present address: Julian M. J. Pietsch, SynthSys—Synthetic and Systems Biology, University of Edinburgh, Edinburgh EH9 3BF, UK.

## References

[1] Ptashne M. A Genetic Switch Phage Lambda Revisited. 2004; Cold Spring Harbor Laboratory Press.

[2] Stokar-Avihail A., Tal N., Erez Z., Lopatina A., Sorek R. Widespread utilization of peptide communication in phages infecting soil and pathogenic bacteria. Cell Host Microbe. 2019; 25:746–755. 31071296 10.1016/j.chom.2019.03.017 PMC6986904

[3] Bednarz M., Halliday J.A., Herman C., Golding I. Revisiting bistability in the lysis/lysogeny circuit of bacteriophage lambda. PLoS One. 2014; 9:e100876. 24963924 10.1371/journal.pone.0100876 PMC4070997

[4] Balázsi G., van Oudenaarden A., Collins J.J., Acar M., Becskei A., van Oudenaarden A., Acar M., Mettetal J.T., van Oudenaarden A., Ambravaneswaran V. et al. Cellular decision making and biological noise: from microbes to mammals. Cell. 2011; 144:910–925. 21414483 10.1016/j.cell.2011.01.030 PMC3068611

[5] Golding I. Single-cell studies of phage λ: hidden treasures under Occam's rug. Annu. Rev. Virol. 2016; 3:453–472. 27482899 10.1146/annurev-virology-110615-042127

[6] Oppenheim A.B., Kobiler O., Stavans J., Court D.L., Adhya S. Switches in bacteriophage lambda development. Annu. Rev. Genet. 2005; 39:409–429. 16285866 10.1146/annurev.genet.39.073003.113656

[7] Herman C., Ogura T., Tomoyasu T., Hiraga S., Akiyama Y., Ito K., Thomas R., D’Ari R., Bouloc P. Cell growth and lambda phage development controlled by the same essential Escherichia coli gene, ftsH/hflB. Proc. Natl. Acad. Sci. U.S.A. 1993; 90:10861–10865. 8248182 10.1073/pnas.90.22.10861 PMC47878

[8] Banuett F., Hoyt M.A., McFarlane L., Echols H., Herskowitz I. hflB, a new Escherichia coli locus regulating lysogeny and the level of bacteriophage lambda cII protein. J. Mol. Biol. 1986; 187:213–224. 2939254 10.1016/0022-2836(86)90229-9

[9] Hoyt M.A., Knight D.M., Das A., Miller H.I., Echols H. Control of phage lambda development by stability and synthesis of cII protein: role of the viral cIII and host hflA, himA and himD genes. Cell. 1982; 31:565–573. 6218885 10.1016/0092-8674(82)90312-9

[10] Murchland I., Ahlgren-Berg A., Priest D.G., Dodd I.B., Shearwin K.E. Promoter activation by CII, a potent transcriptional activator from bacteriophage 186. J. Biol. Chem. 2014; 289:32094–32108. 25294872 10.1074/jbc.M114.608026 PMC4231686

[11] Neufing P.J., Shearwin K.E., Camerotto J., Egan J.B. The CII protein of bacteriophage 186 establishes lysogeny by activating a promoter upstream of the lysogenic promoter. Mol. Microbiol. 1996; 21:751–761. 8878038 10.1046/j.1365-2958.1996.351394.x

[12] Nilsson H., Cardoso-Palacios C., Haggård-Ljungquist E., Nilsson A.S. Phylogenetic structure and evolution of regulatory genes and integrases of P2-like phages. Bacteriophage. 2011; 1:207–218. 23050214 10.4161/bact.1.4.18470 PMC3448106

[13] Christie G.E., Calendar R. Bacteriophage P2. Bacteriophage. 2016; 7081:e1145782. 10.1080/21597081.2016.1145782 PMC4836473 27144088

[14] Roberts J.W., Roberts C.W., Craig N.L. Escherichia coli recA gene product inactivates phage lambda repressor. Proc. Natl. Acad. Sci. U.S.A. 1978; 75:4717–4718. 10.1073/pnas.75.10.4714 PMC336190 368796

[15] Lamont I., Brumby A.M., Egan J.B. UV induction of coliphage 186: prophage induction as an SOS function. Proc. Nat. Acad. Sci. U.S.A. 1989; 86:5492–5496. 10.1073/pnas.86.14.5492 PMC297649 2664785

[16] Shearwin K.E., Brumby A.M., Egan J.B. The tum protein of coliphage 186 is an antirepressor. J. Biol. Chem. 1998; 273:5708–5715. 9488703 10.1074/jbc.273.10.5708

[17] Kobiler O., Rokney A., Friedman N., Court D.L., Stavans J., Oppenheim A.B. Quantitative kinetic analysis of the bacteriophage λ genetic network. Proc. Natl. Acad. Sci. 2005; 102:4470–4475. 15728384 10.1073/pnas.0500670102 PMC549295

[18] Lamont I., Richardson H., Carter D.R., Egan J.B. Genes for the establishment and maintenance of lysogeny by the temperate coliphage 186. J. Bacteriol. 1993; 175:5286–5288. 8349570 10.1128/jb.175.16.5286-5288.1993 PMC205000

[19] Akiyama Y., Ogura T., Ito K. Involvement of FtsH in protein assembly into and through the membrane. I. Mutations that reduce retention efficiency of a cytoplasmic reporter. J. Biol. Chem. 1994; 269:5218–5224. 8106504

[20] Ogura T., Inoue K., Tatsuta T., Suzaki T., Karata K., Young K., Su L.-H., Fierke C.A., Jackman J.E., Raetz C.R.H. et al. Balanced biosynthesis of major membrane components through regulated degradation of the committed enzyme of lipid A biosynthesis by the AAA protease FtsH (HflB) in Escherichia coli. Mol. Microbiol. 1999; 31:833–844. 10048027 10.1046/j.1365-2958.1999.01221.x

[21] Kanehara K., Ito K., Akiyama Y. YaeL (EcfE) activates the sigma E pathway of stress response through a site-2 cleavage of anti-sigma E, RseA. Genes Dev. 2002; 16:2147–2155. 12183368 10.1101/gad.1002302 PMC186437

[22] Akiyama Y., Ito K. Reconstitution of membrane proteolysis by FtsH. J. Biol. Chem. 2003; 278:18146–18153. 12642574 10.1074/jbc.M302152200

[23] Datsenko K.A., Wanner B.L. One-step inactivation of chromosomal genes in Escherichia coli K-12 using PCR products. Proc. Natl. Acad. Sci. U.S.A. 2000; 97:6640–6645. 10829079 10.1073/pnas.120163297 PMC18686

[24] Shearwin K.E., Egan J.B. Establishment of lysogeny in bacteriophage 186. DNA binding and transcriptional activation by the CII protein. J. Biol. Chem. 2000; 275:29113–29122. 10871623 10.1074/jbc.M004574200

[25] Pinkett H.W., Shearwin K.E., Stayrook S., Dodd I.B., Burr T., Hochschild A., Egan J.B., Lewis M. The structural basis of cooperative regulation at an alternate genetic switch. Mol. Cell. 2006; 21:605–615. 16507359 10.1016/j.molcel.2006.01.019

[26] Priest D.G., Cui L., Kumar S., Dunlap D.D., Dodd I.B., Shearwin K.E. Quantitation of the DNA tethering effect in long-range DNA looping in vivo and in vitro using the Lac and λ repressors. Proc. Natl. Acad. Sci. U.S.A. 2014; 111:349–354. 24344307 10.1073/pnas.1317817111 PMC3890862

[27] St-Pierre F., Cui L., Priest D.G., Endy D., Dodd I.B., Shearwin K.E. One-step cloning and chromosomal integration of DNA. ACS Synth. Biol. 2013; 2:537–541. 24050148 10.1021/sb400021j

[28] Sharan S.K., Thomason L.C., Kuznetsov S.G., Court D.L. Recombineering: a homologous recombination-based method of genetic engineering. Nat. Protoc. 2009; 4:206–223. 19180090 10.1038/nprot.2008.227 PMC2790811

[29] Datta S., Costantino N., Court D.L. A set of recombineering plasmids for gram-negative bacteria. Gene. 2006; 379:109–115. 16750601 10.1016/j.gene.2006.04.018

[30] Dodd I.B., Reed M.R., Egan J.B. The Cro‐like Apl repressor of coliphage 186 is required for prophage excision and binds near the phage attachment site. Mol. Microbiol. 1993; 10:1139–1150. 7934863 10.1111/j.1365-2958.1993.tb00983.x

[31] Schubert R.A., Dodd I.B., Egan J.B., Shearwin K.E. Cro's role in the CI Cro bistable switch is critical for lambda's transition from lysogeny to lytic development. Genes Dev. 2007; 21:2461–2472. 17908932 10.1101/gad.1584907 PMC1993876

[32] Palmer A.C., Ahlgren-Berg A., Egan J.B., Dodd I.B., Shearwin K.E. Potent transcriptional interference by pausing of RNA polymerases over a downstream promoter. Mol. Cell. 2009; 34:545–555. 19524535 10.1016/j.molcel.2009.04.018 PMC2697128

[33] Kiehl T.R., Mattheyses R.M., Simmons M.K. Hybrid simulation of cellular behavior. Bioinformatics. 2004; 20:316–322. 14960457 10.1093/bioinformatics/btg409

[34] Bernstein J.A., Khodursky A.B., Lin P., Lin-chao S., Cohen S.N. Global analysis of mRNA decay and abundance in Escherichia coli at single-gene resolution using two-color fluorescent DNA microarrays. Proc. Natl. Acad. Sci. U.S.A. 2002; 99:9697–9702. 12119387 10.1073/pnas.112318199 PMC124983

[35] Goryshin I.Y., Jendrisak J., Les M., Meis R., Reznikoff W.S. Insertional transposon mutagenesis by electroporation of released Tn5 transposition complexes. Nat. Biotechnol. 2000; 18:97–100. 10625401 10.1038/72017

[36] Baba T., Ara T., Hasegawa M., Takai Y., Okumura Y., Baba M., Datsenko K.A., Tomita M., Wanner B.L., Mori H. Construction of Escherichia coli K-12 in-frame, single-gene knockout mutants: the Keio collection. Mol. Syst. Biol. 2006; 2:2006.0008. 10.1038/msb4100050 PMC1681482 16738554

[37] Akiyama Y., Kanehara K., Ito K. RseP (YaeL), an Escherichia coli RIP protease. EMBO J. 2004; 23:4434–4442. 15496982 10.1038/sj.emboj.7600449 PMC526465

[38] Herman C., Thévenet D., D’Ari R., Bouloc P. Degradation of sigma 32, the heat shock regulator in Escherichia coli, is governed by HflB. Proc. Natl. Acad. Sci. U.S.A. 1995; 92:3516–3520. 7724592 10.1073/pnas.92.8.3516 PMC42198

[39] Shearwin K.E., Callen B.P., Egan J.B. Transcriptional interference - a crash course. Trends Genet. 2005; 21:339–345. 15922833 10.1016/j.tig.2005.04.009 PMC2941638

[40] Dodd I.B., Egan J.B. Action at a distance in CI repressor regulation of the bacteriophage 186 genetic switch. Mol. Microbiol. 2002; 45:697–710. 12139616 10.1046/j.1365-2958.2002.03038.x PMC2941640

[41] Svenningsen S.L., Semsey S. Commitment to lysogeny is preceded by a prolonged period of sensitivity to the late lytic regulator Q in bacteriophage λ. J. Bacteriol. 2014; 196:3582–3588. 25092034 10.1128/JB.01705-14 PMC4187692

[42] Zeng L., Skinner S.O., Zong C., Sippy J., Feiss M., Golding I. Decision making at a subcellular level determines the outcome of bacteriophage infection. Cell. 2010; 141:682–691. 20478257 10.1016/j.cell.2010.03.034 PMC2873970

[43] Cui L., Murchland I., Shearwin K.E., Dodd I.B. Enhancer-like long-range transcriptional activation by CI-mediated DNA looping. Proc. Natl. Acad. Sci. U.S.A. 2013; 110:2922–2927. 23382214 10.1073/pnas.1221322110 PMC3581938

[44] St-Pierre F., Endy D. Determination of cell fate selection during phage lambda infection. Proc. Natl. Acad. Sci. U.S.A. 2008; 105:20705–20710. 19098103 10.1073/pnas.0808831105 PMC2605630

[45] Herskowitz I., Hagen D. The lysis-lysogeny decision of phage lambda: explicit programming and responsiveness. Annu. Rev. Genet. 1980; 14:399–445. 6452089 10.1146/annurev.ge.14.120180.002151

[46] Kobiler O., Rokney A., Oppenheim A.B. Phage lambda CIII: a protease inhibitor regulating the lysis-lysogeny decision. PLoS One. 2007; 2:e363. 17426811 10.1371/journal.pone.0000363 PMC1838920

[47] Rokney A., Kobiler O., Amir A., Court D.L., Stavans J., Adhya S., Oppenheim A.B. Host responses influence on the induction of lambda prophage. Mol. Microbiol. 2008; 68:29–36. 18298445 10.1111/j.1365-2958.2008.06119.x PMC2327240

[48] Avlund M., Krishna S., Semsey S., Dodd I.B., Sneppen K. Minimal gene regulatory circuits for a lysis-lysogeny choice in the presence of noise. PLoS One. 2010; 5:e15037. 21188148 10.1371/journal.pone.0015037 PMC3004801

[49] Wang H., Dodd I.B., Dunlap D.D., Shearwin K.E., Finzi L. Single molecule analysis of DNA wrapping and looping by a circular 14mer wheel of the bacteriophage 186 CI repressor. Nucleic Acids Res. 2013; 41:5746–5756. 23620280 10.1093/nar/gkt298 PMC3675496

