# Supplementary material for: Instability of CII is needed for efficient switching between lytic and lysogenic development in bacteriophage 186
Source: Nucleic Acids Res. 2020 Nov 19;48(21):12030–41. doi: 10.1093/nar/gkaa1065 (PMC7708051; doi:10.1093/nar/gkaa1065)
Supplement: gkaa1065_Supplemental_File [file gkaa1065_supplemental_file.pdf]

Supplementary Data

Mathematical model of the bacteriophage 186 switch

Our model of the bacteriophage 186 switch region attempts to capture the most important known regulatory interactions that impact on the decision between lytic or lysogenic development upon infection of a bacterium. The decision made in a single cell is thought to be stochastic as a result of fluctuations at the molecular scale. Whilst much is understood about the average behaviour of the regulatory network, the primary sources of noise for bacteriophage 186 are unknown. Nonetheless, it is likely that stochastic production and degradation of the transcripts play a significant role, so for simplicity we limit the stochastic treatment to just those reactions, treating the remaining reactions deterministically. Custom software for simulation of hybrid stochastic-deterministic models using the 'next reaction' variant of Gillespie's algorithm (1) was written in C++ with an API written for R to facilitate model specification. Software is available on request.

The reactions included in the model are listed in Supplementary Table 1 and describe regulation of the lytic (*pR*), lysogenic (*pL*) and establishment (*pE*) promoters by the products of the lytic transcript (Apl and CII) and by the product of the lysogenic transcript (CI). Variants from the wildtype phage were simulated by simple adjustments to the parameters as listed in Supplementary Table 2. The trans-*pE-lacZ* reporter strains were modelled as a CI^{-} network, taking the hazard for production of *pE* transcripts as proportional to LacZ activity, with equality when both are normalised by maximum promoter activity. In Figure 7 (main text), the rate of production/loss of CI was calculated directly at each time point as the value of equation 8 in Supplementary Table 1. Regulation of *pR* and *pL* activity by the repressor CI is non-trivial, involving both multimerisation of the transcription factor at the DNA and transcriptional interference between the promoters. A detailed model of both processes has previously been described (2)(3) and was fit to population measurements of promoter activity for inducible concentrations of CI (4). For simplicity and clarity in this context, however, we chose instead to fit that data using Hill equations in the concentration of CI (equations 3 and 5 in Supplementary Table 1). The best least squares fit to the experimental data is shown in Supplementary Figure 1.

Estimates for many of the other parameters have been determined previously. The degradation rate of CII was from (5), and the rates of loss of CI and Apl were assumed to be dilution limited. Promoter firing rates for *pR* and *pL* were from (2), with that for *pE* being estimated similarly by comparing promoter strengths. CII activation of the *pE* promoter was modelled as a Hill function with parameters fit to *in vitro* transcription data (5). Apl repression was assumed to act equally on *pR* and *pL*, with parameters from a fit of 7-site gel shift data (6).

The protein translation rates and transcript degradation rates could not be determined from elsewhere. For the translation rates, we noted that over a broad range of parameters, simulations of the steady-state behaviour of lysogenic phage (corresponding to the variants in main text Fig. 5A) and of the *trans-pE* reporter strains (main text Fig. 6A) showed average behaviour that closely matched that of the equivalent deterministic model. As such, we were able to fit these parameters to reproduce the experimental data (Fig. 7A) by solving the equivalent deterministic model at steady state. A weighted least squares scheme was used in which residual errors were generally assumed to scale with the magnitude of the model output. Specifically, the following equation was minimised:

$$R^2 = \sum_{l \in L} \left(\frac{[CI]_t^{(l)}}{[CI]_t^{(expt)}} - 1 \right)^2 + \sum_{t \in T} \left(\frac{[Z]^{(t)}}{[Z]^{(expt,t)}} - 1 \right)^2, \qquad \textbf{(1)}$$

where $[CI]_t^{(l)}$ is the average total concentration of CI in each of the three lysogen variants, and $[Z]^{(t)}$ and $[Z]^{(expt,t)}$ are, respectively, the average values of *trans-pE* activity (normalised by maximum *pE* activity) for each of the eight *trans-pE* reporter variants. We had no data with which to fit the transcript degradation rates, so made them all equal, choosing a value typical of mRNA half-lives observed in *E. coli* (7). We note that within a biologically feasible range, the transcript degradation rates had little influence on the model outputs of interest.

Supplementary Table 1: Reactions included in the mathematical model of the bacteriophage 186 switch region.

Gene network component	Mathematical description	Parameter values
Stochastically modelled inhomogeneous Poisson processes with time-varying hazard $\lambda(t)$		
**1.** Production of *pE* transcripts. Each event yields one *pE* transcript (*pEt*).	$\lambda(t) = r_{pE} \times \frac{\left(\frac{[CII]_t}{EC50_{pE}}\right)^{h_{pE}}}{1 + \left(\frac{[CII]_t}{EC50_{pE}}\right)^{h_{pE}}}$	$r_{pE} = 0.030$ s^{-1}<br>$EC50_{pE} = 12$nM<br>$h_{pE} = 1.17$
**2.** Degradation of *pE* transcripts. Each event destroys one *pEt.*	$\lambda(t) = \frac{\log 2}{\langle lifetime_{pEt}\rangle}[pEt]_t$	$\langle lifetime_{pEt}\rangle = 300$ s
**3.** Production of *pL* transcripts. Each event yields one *pL* transcript (*pLt*).	$\lambda(t) = frac(Apl) \times r_{pL} \times \left(B_{pL(Act)} + \frac{\left(\frac{[CI]_t}{EC50_{pL(Act)}}\right)^{h_{pL(Act)}}}{1 + \left(\frac{[CI]_t}{EC50_{pL(Act)}}\right)^{h_{pL(Act)}}}\right) \times \frac{1}{1 + \left(\frac{[CI]_t}{EC50_{pL(Rep)}}\right)^{h_{pL(Rep)}}}$	$r_{pL} = 0.0056$ s^{-1}<br>$B_{pL(Act)} = 0.12$<br>$EC50_{pL(Act)} = 144$ nM<br>$h_{pL(Act)} = 1.00$<br>$EC50_{pL(Rep)} = 530$ nM<br>$h_{pL(Rep)} = 1.23$
**4.** Degradation of *pL* transcripts. Each event destroys one *pLt.*	$\lambda(t) = \frac{\log 2}{\langle lifetime_{pLt}\rangle}[pLt]_t$	$\langle lifetime_{pLt}\rangle = 300$ s
**5.** Production of *pR* transcripts. Each event yields one *pR* transcript (*pRt*).	$\lambda(t) = r_{pR} \times \frac{1}{1 + \left(\frac{[CI]_t}{EC50_{CI}}\right)^{h_{CI}}} \times frac(Apl)$	$r_{pR} = 0.056$ s^{-1}<br>$EC50_{CI} = 3.2$ nM<br>$h_{CI} = 0.73$
**6.** Degradation of *pR* transcripts. Each event destroys one *pRt.*	$\lambda(t) = \frac{\log 2}{\langle lifetime_{pRt}\rangle}[pRt]_t$	$\langle lifetime_{pRt}\rangle = 300$ s
Deterministically modelled processes		
**7.** Fractional inhibition of *pR*-*pL* by Apl.	$frac(Apl) = \frac{1}{1 + \left(\frac{[Apl]}{EC50_{Apl}}\right)^{h_{Apl}}}$	$EC50_{Apl} = 265$ nM<br>$h_{Apl} = 2.8$
**8.** Production/degradation of CI from *pL* and *pE* transcripts.	$\frac{d[CI]}{dt} = \rho_{CI}([pLt]_t + [pEt]_t) - D_{CI}[CI]_t$	$\rho_{CI} = 0.56$ nM.s^{-1}<br>$D_{CI} = 3.9 \times 10^{-4}$ s^{-1}
**9.** Production of Apl from *pR* transcripts.	$\frac{d[Apl]}{dt} = \rho_{Apl}[pRt]_t - D_{Apl}[Apl]_t$	$\rho_{Apl} = 5.6 \times 10^{-3}$ nM.s^{-1}<br>$D_{Apl} = 3.9 \times 10^{-4}$ s^{-1}
**10.** Production of CII from *pR* transcripts.	$\frac{d[CII]}{dt} = \rho_{CII}[pRt]_t - D_{CII}[CII]_t$	$\rho_{CII} = 4.5 \times 10^{-4}$ nM.s^{-1}<br>$D_{CII} = 4.4 \times 10^{-3}$ s^{-1}

Supplementary Table 2: Parameter adjustments used to specify variants of the 186 switch genetic circuit.

Simulation variant	Parameter alteration(s) from wildtype
CI^{-}	$\rho_{CI} = 0$
Apl^{-}	$\rho_{Apl} = 0$
CII^{-}	$\rho_{CII} = 0$
CII145	$D_{CII} = 3.9 \times 10^{-3}\ \mathrm{s}^{-1}$
pE^{-}	$\rho_{CII} = 0$
P2-like network (1.0x *pL*)	$\rho_{CII} = 0, EC50_{pL(Rep)} = 570\mathrm{nM}$
P2-like network (1.4x *pL*)	$\rho_{CII} = 0, EC50_{pL(Rep)} = 390\mathrm{nM}$
P2-like network (1.8x *pL*)	$\rho_{CII} = 0, EC50_{pL(Rep)} = 300\mathrm{nM}$

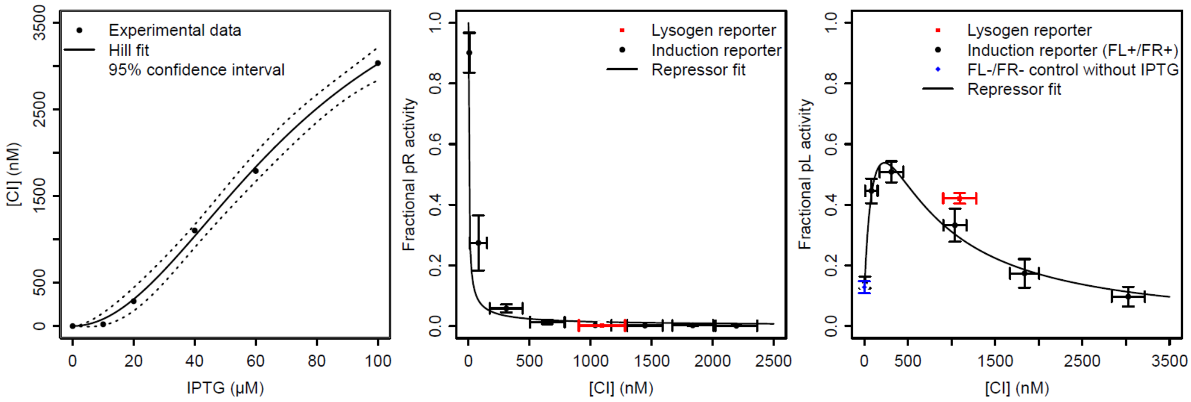

Supplementary Figure 1: Transcriptional interference and CI repression at *pL* and *pR* can be approximated using a combination of Hill functions. The left panel shows a Hill fit for protein induction ultra-sensitivity, relating inducer concentrations to concentrations of CI measured by Western blot (4). The middle and right panels show reporter data (4) for the switch region of bacteriophage 186 at the inducer concentrations shown in the left panel. Red squares indicate experimental measurements of promoter activity at lysogenic concentrations of CI. The black line shows the fit of the model.

REFERENCES

1. Kiehl,T.R., Mattheyses,R.M. and Simmons,M.K. (2004) Hybrid simulation of cellular behavior. *Bioinformatics*, **20**, 316–322.

2. Sneppen,K., Dodd,I.B., Shearwin,K.E., Palmer,A.C., Schubert,R.A., Callen,B.P. and Egan,J.B. (2005) A mathematical model for transcriptional interference by RNA polymerase traffic in Escherichia coli. *J. Mol. Biol.*, **346**, 399–409.

3. Dodd,I.B., Shearwin,K.E. and Sneppen,K. (2007) Modelling transcriptional interference and DNA looping in gene regulation. *J. Mol. Biol.*, **369**, 1200–1213.

4. Dodd,I.B. and Egan,J.B. (2002) Action at a distance in CI repressor regulation of the bacteriophage 186 genetic switch. *Mol. Microbiol.*, **45**, 697–710.

5. Murchland,I., Ahlgren-Berg,A., Priest,D.G., Dodd,I.B. and Shearwin,K.E. (2014) Promoter activation by CII, a potent transcriptional activator from bacteriophage 186. *J. Biol. Chem.*, **289**.

6. Cutts,E.E., Barry Egan,J., Dodd,I.B. and Shearwin,K.E. (2020) A quantitative binding model for the Apl protein, the dual purpose recombination-directionality factor and lysis-lysogeny regulator of bacteriophage 186. *Nucleic Acids Res.*, 10.1093/nar/gkaa655.

7. Bernstein,J.A., Khodursky,A.B., Lin,P., Lin-chao,S. and Cohen,S.N. (2002) Global analysis of mRNA decay and abundance in Escherichia coli at single-gene resolution using two-color fluorescent DNA microarrays. *Proc Natl Acad Sci U S A*, **99**, 9697–9702.
